# Drought patterns: their spatiotemporal variability and impacts on maize production in Limpopo province, South Africa

**DOI:** 10.1007/s00484-022-02392-1

**Published:** 2022-12-07

**Authors:** Nicole Costa Resende Ferreira, Reimund Paul Rötter, Gennady Bracho-Mujica, William C. D. Nelson, Quang Dung Lam, Claus Recktenwald, Isaaka Abdulai, Jude Odhiambo, Stefan Foord

**Affiliations:** 1 Tropical Plant Production and Agricultural Systems Modelling (TROPAGS), Georg-August-Universität Göttingen, Grisebachstraße 6, 37077 Göttingen, Germany; 2 Kasisi Agricultural Training Center (KATC), Kasisi Mission, Farm 591, Lusaka, Zambia; 3 Department of Soil Science, University of Venda, Thohoyandou, 0950 South Africa; 4 Department of Zoology, University of Venda, Thohoyandou, 0950 South Africa

**Keywords:** Climate change, Droughts, South Africa, Crop modeling, Maize

## Abstract

Due to global climate change, droughts are likely to become more frequent and more severe in many regions such as in South Africa. In Limpopo, observed high climate variability and projected future climate change will likely increase future maize production risks. This paper evaluates drought patterns in Limpopo at two representative sites. We studied how drought patterns are projected to change under future climatic conditions as an important step in identifying adaptation measures (e.g., breeding maize ideotypes resilient to future conditions). Thirty-year time horizons were analyzed, considering three emission scenarios and five global climate models. We applied the WOFOST crop model to simulate maize crop growth and yield formation over South Africa’s summer season. We considered three different crop emergence dates. Drought indices indicated that mainly in the scenario SSP5-8.5 (2051–2080), Univen and Syferkuil will experience worsened drought conditions (DC) in the future. Maize yield tends to decline and future changes in the emergence date seem to impact yield significantly. A possible alternative is to delay sowing date to November or December to reduce the potential yield losses. The grain filling period tends to decrease in the future, and a decrease in the duration of the growth cycle is very likely. Combinations of changed sowing time with more drought tolerant maize cultivars having a longer post-anthesis phase will likely reduce the potential negative impact of climate change on maize.

**Supplementary Information:**

The online version contains supplementary material available at 10.1007/s00484-022-02392-1.

## Introduction

Droughts affect different regions globally, with a range of negative impacts affecting multiple socioeconomic and environmental sectors, including agriculture (Vicente-Serrano 2006; Ferreira et al. 2021a), water resources (Ferreira and Chou 2018; Ferreira et al. 2021b), and forestry (Copenheaver et al. 2011), among others. Due to global climate change, droughts are likely to become more frequent and more severe in many regions (Dai 2011), as a consequence of the projected global warming with changes in circulation patterns (e.g., Kornhuber et al. 2019), increased evapotranspiration, changes in rainfall patterns, accelerated hydrological cycle with increased rainfall intensity, etc. (Drumond et al. 2019; Fischer and Knutti 2014; Intergovernmental Panel on Climate Change (IPCC) 2019; Lobell et al. 2013). High temperatures are expected to result in higher water deficits during the summer season, leading to decreased soil moisture and more frequent and severe agricultural droughts (Adams and Peck 2009; Park et al. 2018).

Large-scale droughts have occurred worldwide at different times throughout historical record (Dai 2011; Trnka et al. 2018), yet the damage has increased substantially in recent decades (Moravec et al. 2021). In arid and semi-arid areas of southern Africa, droughts are common and frequent (Park et al. 2018; Meza et al. 2021; Mahlalela et al. 2020) causing significant economic losses (Vogel et al. 2000) and increasing food insecurity in the region (Verschuur et al. 2021). Since 1970, Southern Africa has observed more intense, widespread and more extended droughts (Richard et al. 2001; Burls et al. 2019). In this context, it is important to unravel the spatiotemporal patterns and severity of drought at different scales to support the design and adjustment of climate change mitigation and adaptation measures.

The agriculture sector depends on climate to guarantee crop productivity, profitability, and quality. Lobell et al. (2008) concluded that agricultural production will mainly be negatively affected by climate change and will impede the ability of many regions to achieve the necessary gains for future food security, as was also recently found for the main wheat producing and exporting regions worldwide (Trnka et al. 2019). In southern Africa, maize is predominantly grown in smallholder farming systems, where over 90% of the production systems are rainfed; and also, the maize cultivated by commercial farmers in South Africa is mainly rainfed (Bationo and Waswa 2011). Smallholder maize farming systems in the dry savanna areas, as found in Limpopo (Rötter et al. 2021), are particularly vulnerable to climate variability and change (Adger et al. 2007; Cairns et al. 2013; Conway et al. 2015). This could have a huge impact on local food security due to the importance of these areas to the agricultural sector. While many studies show that climate change will increase drought frequency and severity, the direction and extent of these changes and related crop yields depend on the region and season. For this reason, the use of different drought metrics might be needed to provide robust estimates of related risks (Cook et al. 2020).

This paper aims to study drought patterns in the Limpopo region (South Africa) and evaluate their spatiotemporal patterns and how these are likely to change under future climatic conditions to signal potential repercussions on crop yields. In particular, we will look at 30-year time horizons and consider different emissions scenarios and global climate models. The other important and closely related objective is how drought may potentially affect maize crop production in two representative sites in Limpopo, with contrasting conditions. To quantify climate change’s impact on maize development and yield, we applied the crop growth simulation model WOFOST (Boogaard et al. 1998).

## Materials and methods

### Study area and maize climatic requirements

**Fig. 1:**
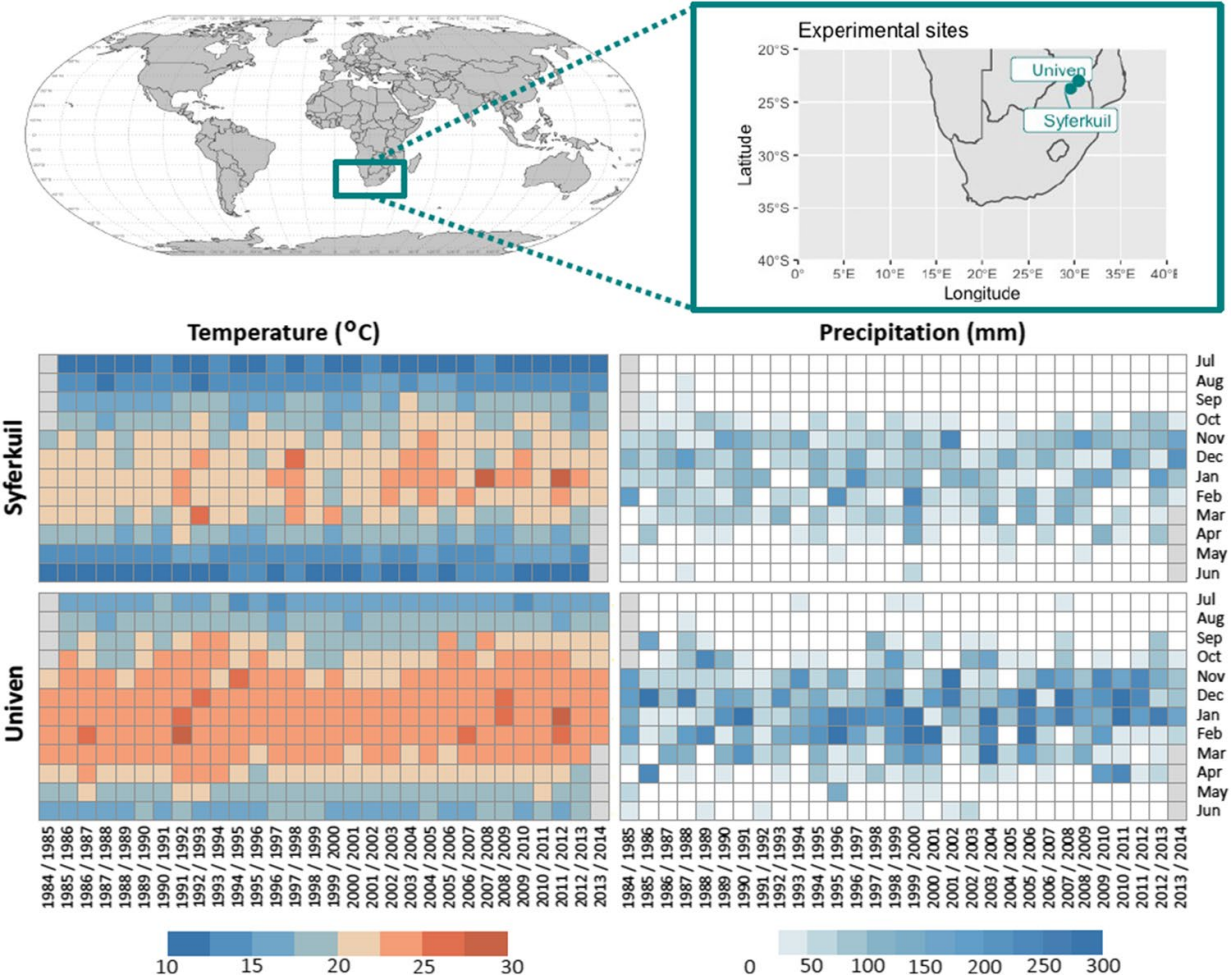
Experimental sites Syferkuil and Univen in Limpopo, SA. Total precipitation (mm) and mean temperatures (°C) monthly climatology (period: 1984–2014). Missing values are shown in gray

The study area comprises parts of the Limpopo province, South Africa (SA). This region is known as one of the hottest provinces in the country (Kruger and Shongwe 2004), with frequent and severe droughts due to high temperatures and unreliable rainfall (Maponya and Mpandeli 2012; Maposa et al. 2021). The region presents mostly a subtropical climate, with a contrasting environment favorable for the cultivation of grain crops, tropical fruits, and vegetables. We focused this study on two sites: Univen and Syferkuil (Fig. 1). These sites were chosen due to the contrasting environmental conditions (i.e., soil and climate characteristics) and long-term data availability.

The interannual variability of total accumulated precipitation per year is higher at Univen (Online Resource 1) than at Syferkuil. In both sites, there is a clear seasonal pattern in precipitation, from October until March, but Univen is a warmer site. In Syferkuil, there is a distinct increase in air temperature from 2003 onwards, especially in January.

In SA, several circulation phenomena influence climate variability, including El Niño Southern Oscillation (ENSO) phenomenon—with contrasting impacts associated with El Niño and La Niña phases (Reason and Jagadheesha 2005; Gizaw and Gan 2017). As a general rule, the El Niño phase tends to lead to drier conditions, whereas the La Niña phase tends to lead to wetter conditions (Reason and Jagadheesha 2005; Phillips et al. 1998; Nicholson and Kim 1997; Janowiak 1988). The spatial extent of drought-prone regions of SA may increase in the future due to an increase in the frequency of El Niño episodes under a warmer climate (Diaz et al. 2001; Perry et al. 2017; Cai et al. 2015; Power et al. 2013). Pomposi et al. (2018) verified that strong and moderate-to-weak El Niño events tend to increase dry days in southern Africa. The same authors concluded that the likelihood of southern Africa receiving less than average precipitation is approximately 80% for strong El Niño events compared to just over 60% for moderate-to-weak El Niño events. A detailed comparison of the precipitation patterns of average years with El Niños and strong El Niños is given in the supplementary material (Online Resources 2 and 3). In years with strong El Niño events, especially the south-eastern region become drier than normal, and in the north-eastern region, strong El Niños have an opposite effect. Therefore, when looking at the country’s average yield, the ENSO impact for some regions may be masked, leading to little overall effect on yield (as shown, e.g., in Mozambique, Angola, Zambia). In other countries, such as South Africa and Botswana, where we observe drought patterns exclusively in years of strong El Niños, the relationship between drought and yield is more consistent on a year-to-year basis.

Regarding maize production in SA, the Limpopo region plays an essential role. A great share of its maize production (62%) is provided by smallholder farmers (LEDET, 2016). According to Agbiz (2016), maize is the most cultivated grain crop in SA, followed by soybeans, wheat, sunflower, and sugar cane (FAOSTAT 2019). The average maize yield (t.ha^−1^) from 1990 to 2019 for Limpopo and SA, and their relationship with El Niño can be found at Online Resource 4. In the last decade, the average commercial yield in Limpopo exceeded the average values in SA, emphasizing the region’s importance in the national agricultural development. The climate conditions in Limpopo and especially the low mean annual precipitation are known as factors limiting yields attainable under rainfed conditions (Conway et al. 2015; Trambauer et al. 2014).

Limpopo is one of those areas of SA frequently prone to drought events (Dlamini 2013). The current climate variability as observed in Limpopo and the expected future climatic change may impose higher future risks to crop production. The climate vulnerability in SA is also emphasized by the fact that most maize production is rainfed, with less than 10% produced under irrigation (Baloyi 2011). The location and time of the year/length of the growing season are critical factors that determine the potential impacts of climate change on crop production (Gbetibouo and Hassan 2005). Each crop has climatic requirements including crop water requirements, which mainly depend on the crop’s genetic characteristics, stage of growth, and duration of the growth cycle.

### Climate projections

Projections from Phase Six of the Coupled Model Intercomparison Project (CMIP6) and the Inter-Sectoral Impact Model Intercomparison Project (ISI-MIP) provide climate scenarios based on different Shared Socio-Economic Pathways (SSP) (O’Neill et al. 2014, 2020). These climate scenarios can be used to investigate the implications of long-term climatic changes for designing robust policies in an environment of interacting complex systems and uncertainty (Hall et al. 2016; Harrison et al. 2015; O’Neil et al. 2014).

For this study, we selected the scenarios SSP1-2.6, SSP3-7.0, and SSP5-8, which can be considered optimistic, intermediate, and pessimistic climate change scenarios, respectively. We used BIAS-adjusted precipitation data (Lange 2019) for the historical and future periods. Climate change projections were divided into two 30-year time-slices, from 2021 to 2050 and 2051 to 2080. The climate models were selected according to their availability: IPSL-CM6A-LR, GFDL-ESM4, MPI-ESM1-2-HR, MRI-ESM2-0, and UKESM1-0-LL. The models have a horizontal resolution of 0.5° × 0.5°, and for convenience, they will be named as IPSL, GFDL, MPI, MRI, and UKESM, respectively. To evaluate the climate models’ performance, we used simulated historical climate data (1981–2010) and observed climate data (1984 to 2014) and calculated the root mean squared error (RMSE) and the mean bias error (MBE). Different meteorological drought indices were calculated, and an ensemble mean model was created for assessing the temporal and spatial patterns of drought.

### Drought analyses

Masih et al. (2014) presented a review of droughts on the African continent from 1900 to 2013, indicating that droughts have become more frequent, intense, and widespread during the last 50 years. In SA, droughts occur often and during different times of the year in all climatic zones, with different intensity, spatial extent, and duration (Rouault and Richard 2003). In the Limpopo province, drought imposes a considerable risk since large parts of the province have a semi-arid climate with low, erratic rainfall (Maponya and Mpandeli 2012).

Several indices are commonly used as proxies to capture different drought patterns based on climatic information. Those indices were developed to characterize drought considering different approaches based on its magnitude, duration, frequency, and intensity (Heim 2000, 2002; Vicente-Serrano et al. 2010; Dai 2011; Edossa et al. 2016; Rouault and Richard 2003).

In this study, six indices were selected to represent different drought conditions: PRCPTOT (total precipitation accumulated per month, mm), DD (dry days: the number of days without precipitation), LDP (longest dry period: the number of consecutive days without precipitation), LWP (longest wet period: number of consecutive days with precipitation), RX5D (maximum consecutive 5-day precipitation within a month, mm), and SPI (standardized precipitation index for classification of drought severity).

Indices were calculated for the maize growing period in the study area (i.e., from October to March). This period was chosen since the main maize planting time is between mid-October and mid-December (Matimolane 2018). Each index can help to understand drought patterns in a different way and thus jointly provide the basis for the design of effective adaptation/mitigation measures.

### Crop simulation modelling

Climate extremes, such as drought, have several impacts on crop performance, affecting among others, the sowing dates, nutrient management practices, and eventually the actual yield obtained. In this context, process-based crop models are widely used tools for predicting crop growth and yield on the basis of crop characteristics and their interaction with prevailing weather and soil conditions. These tools can support current and future agricultural field management and national decision-making, e.g., the widely applied modelling platforms APSIM (Keating et al. 2003), DSSAT (Jones et al. 2003), and WOFOST (Van Ittersum et al. 2003). It is expected that future changes in temperature and precipitation regimes will be directly reflected by changes in crop yields all over the world, whereby negative yield impacts are likely to be prevalent in many regions, including most African countries (Abraha and Savage 2006; Porter et al. 2014; Waha et al. 2013).

Among the crop simulation models that have been applied in Africa, we chose the World Food Studies (WOFOST 7.1) model for simulating daily crop growth and spring maize yield under rainfed conditions in SA under different climate change scenarios (Ma et al. 2013; Boogard et al. 2013, 1998; de Wit et al. 2019). The WOFOST model simulates the phenological development of different crops, from emergence to maturity, considering the crop genetic properties and environmental conditions (Hadiya et al. 2018). It comprises different processes such as phenological development, light interception, CO2 assimilation, transpiration, respiration, partitioning of assimilates to the various organs, dry matter, and yield formation (Boogard et al. 1998; Hadiya et al. 2018). The WOFOST model has been applied and is continuously being evaluated and extended for different crops all over the world (Dobermann et al. 2000; Palosuo et al. 2011; Rötter et al. 2012; Cheng et al. 2016; de Wit et al. 2019) . Previous calibration and validation of the WOFOST model for different regions in Africa can be found at Liu (2015), Wolf et al. (2015), Kassie et al. (2014), Rötter and van Keulen (1997), and Ogutu et al. (2018).

WOFOST requires as input data: daily weather, soil information, and crop characteristics. Among the input data needed are station name, latitude, longitude, altitude, minimum temperature, maximum temperature, hours of bright sunshine duration or global radiation, wind speed at 2 m, rainfall amount, and vapor pressure. The soil characteristics required are soil texture and soil moisture volumetric fraction at field capacity (cm^3^.cm^−3^), at permanent wilting point (cm^3^.cm^−3^), and at saturation (cm^3^.cm^−3^). To calibrate the model for a given crop cultivar, it is required to have information about crop phenology, maximum leaf area index (LAImax), biomass partitioning pattern, final biomass, and grain yield; if possible, data on soil moisture content in the root zone at some point in time will allow us to cross-check soil water balance calculations.

Sowing dates and crop emergence can have a considerable impact on crop performance. Usually, farmers plant flexibly within a sowing window depending on the location. We considered three different dates of crop emergence, 15 October (julian day 288), 15 November (319), and 15 December (349), based on crop calendars for the given regions. The sites are classified as sandy clay loams, and the soil properties were taken from the recent high resolution (30 m × 30 m) digital soil map iSDA (2020). Online Resource 5 describes the full set up of the model runs applied in this research.

Besides using different emergence dates, we used different climate scenarios to identify how climate change may affect maize production in the region. We used data from the climate models considering the historical period (1981–2010) and the future projections SSP1-2.6, SSP3-7.0, and SSP5-8.5 (2021–2050, 2051–2080). We simulated potential yield (Yp) and water-limited yield (Ywl). We evaluated the water-limited yield (t.ha^−1^), yield gap (calculated as the difference between potential yield and water-limited yield) (t.ha^−1^), grain filling period (defined as the period between the day of flowering and the harvest) (days), and cycle duration (days). Model annual outputs were evaluated to understand how climate change, and more specifically drought occurrence will affect maize yield year-to-year variability.

## Results and discussion

### Drought climatology

We examined the drought climatology using precipitation data in the Limpopo province to verify the variations of long-term annual drought patterns according to historical observations as well as historical weather simulations. Such analysis is essential to identify the years with drought conditions (DC) and identify differences between the two experimental sites (Online Resource 6).

The highest errors in the models are identified for the climate zone represented by Univen site, which has higher amounts of rainfall than the climate zone represented by Syferkuil. In Univen, models underestimate precipitation, as seen from the accumulated precipitation (PRCPTOT) and maximum consecutive 5-day precipitation (RX5D) indices in the NDJF season. In 1999/2000, at Univen, the PRCPTOT index showed a big difference between observed and simulated data. While observations indicated a high value (436.5 mm) of monthly accumulated precipitation (NDJF season), the climate models used in this study were unable to represent this well. A similar pattern is also seen in the RX5D index. The PRCPTOT values in November and December of 1999 were below long-term climatic means (0 and 69 mm, respectively), yet, in January and February of 2000, the highest values recorded in the subregion were observed, with accumulated precipitation of 783 and 894 mm, respectively. These high amounts of precipitation resulted in a disastrous flooding, causing losses of human lives, as well as considerable economic losses (Khandlhela and May 2006). Recktenwald (2019) reported that the southern summer season of 1991/1992 was dry, with droughts occurring in Limpopo. The results agree with the observed drought record, which indicates major droughts in 1991–1992 and 2004–2005 (Walz et al. 2020; Meza et al. 2021). At Univen, model simulations show an increase in DD in 1999, while according to observed data, there was a decrease in DD. At Syferkuil, climate model simulations underestimated DD. The longest wet period index (LWP) shows great variations among the models (e.g., for 1995 and 2005); hence, it appears that the climate model ensemble cannot adequately capture observed extremes. Regarding the longest dry period index (LDP), at Syferkuil, the models indicated low LDP values, while the observations showed high values in NDJF. The standardized precipitation index (SPI) index also shows great variations across the years and between both sites, which can have several implications for agricultural production. Similar results were found by Manatsa et al. (2010) for Zimbabwe.

The definition of drought conditions (DC) for each site was calculated based on specific quantiles (q10 and q90, Online Resource 7). According to the historical simulations, the most critical values are not associated with a specific month or associated with one region only. However, the months of October and March seem to be very problematic in both areas. The driest conditions of accumulated monthly precipitation (PRCPTOT) are found at the Univen site, mainly in October (q10 is 28.4 mm). October is also the month with the lowest accumulated precipitation values in 5 days (q10 is 17.2 mm) and the shortest wet period (q10 is 2 days). October and November are usually the beginning of the rainy season, and droughts in November can be reflected in delayed sowing, as changing planting dates is a common drought adaptation measure applied by farmers in the Limpopo region (May, 2019). At the Univen site, March appears to be the month with the worst drought conditions when considering the number of days without rainfall (DD) and consecutive days without rainfall (LDP).

At Syferkuil, October presents DC due to the low accumulated rainfall in November (q10 PRCPTOT is 35.7 mm), low accumulated rainfall in 5 days (q10 RX5DAY is 19.5 mm), and low values of the longest wet period (2.2 days). October is also the month with the most critical DC, with the highest dry days (26 days) and consecutive dry days (17.2 days). February, on the other hand, presents high values of accumulated rainfall. In general, the Univen site presented more severe DC than the Syferkuil site.

### Evaluation of observed against modelled climate data

**Fig. 2:**
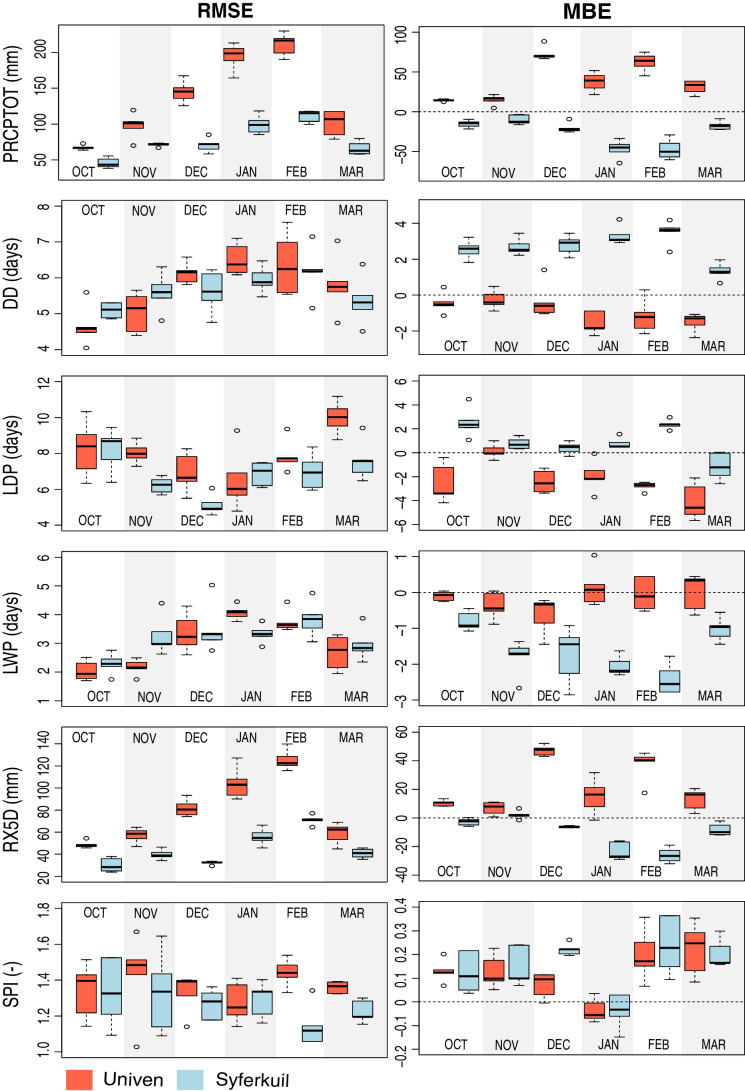
RMSE and MBE for Univen (red) and Syferkuil (blue), calculated based on historical simulations and observed data (1984–2014). The boxplots indicate the errors of the different models for each index and each month (from October to March) considered in this analysis. PRCPTOT represents the total precipitation (mm), DD is the number of dry days (days), LDP is the longest dry period (days), LWP is the longest wet period (days), RX5D is the maximum consecutive 5-day precipitation (mm), and SPI is the standardized precipitation index (-)

Figure 2 indicates the RMSE and the MBE for October to March for the six drought indices. The PRCPTOT index showed positive MBE at Univen and negative at Syferkuil, which indicates the overestimation of the index by climate models at Univen and underestimation at Syferkuil. The largest RMSE occurs in Univen in February. The RX5D also has a higher RMSE for the Univen site, whereby January and February are the months with the biggest errors. The MBE of the DD index indicates underestimation at Univen and overestimation at Syferkuil. The smallest RMSE in the LDP index occurs at Syferkuil, while for the LWP, the smallest errors occur at Univen. Considering the SPI index, the smallest MBE is found for January at Univen and for February at Syferkuil.

In general, the RMSE is lower for the ensemble mean compared to the individual models. The same result is found for MBE, which tends to approach zero when using the ensemble. This observation confirms the suggestion that multi-member ensemble tend to compensate for errors (Rötter et al. 2011; Wallach et al. 2016). The patterns of underestimation or overestimation depend on the index studied, the model evaluated, and the climatic “subregion.” Variations in errors among the models indicate projections’ uncertainty, which is reflected in the ensemble. As shown in Fig. 2, the climate models exhibit differences reflected in model ensemble prediction uncertainty, as they are different numerical system realizations with different types and patterns of errors (Wallach et al. 2016). To reduce uncertainties in the predictions, we applied the mean values of multimember model ensembles in the next steps of this analysis to obtain more robust results (Martre et al. 2015).

### Historical and future drought patterns

**Fig. 3:**
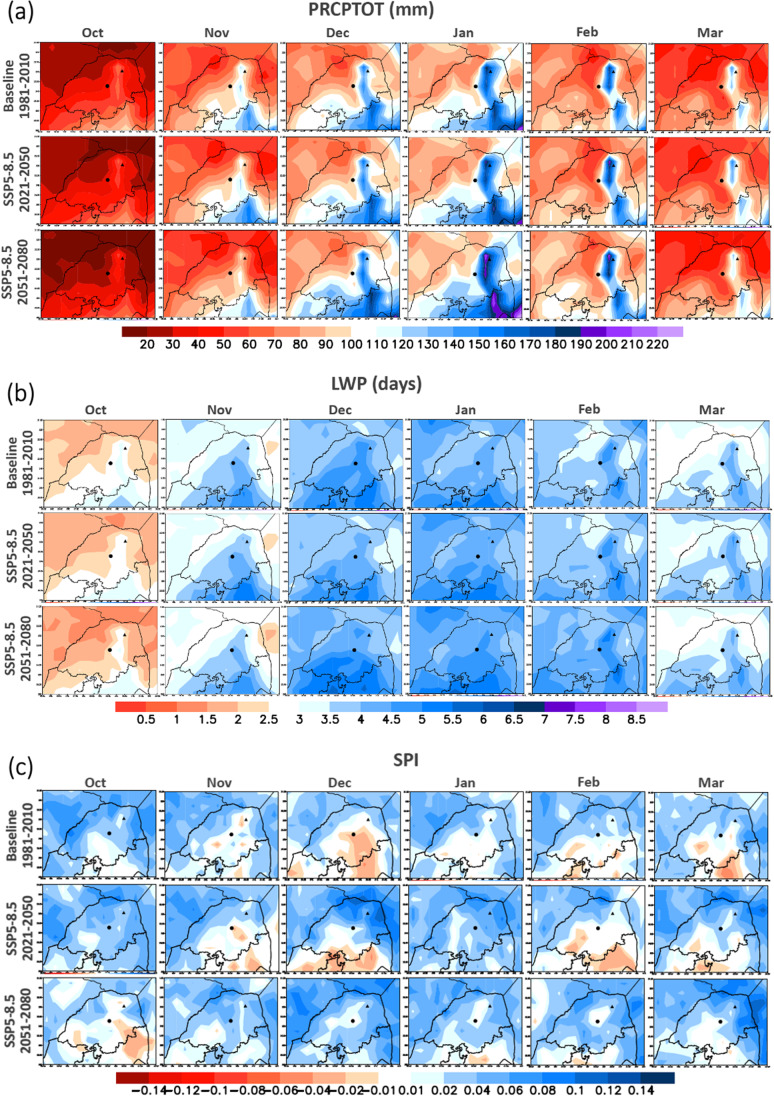
Ensemble 30-year average of the index: a PRCPTOT, b LWP, and c SPI in the Limpopo province for historical simulation (baseline) and future scenario SSP5-8.5 (2021–2050, 2051–2080). Sites are represented with a black circle (Syferkuil), and a black triangle (Univen)

To study drought patterns and their shifts in the future, we assessed indices across the Limpopo region. We evaluated the indices using the 30-year averages of the baseline (1981–2010) and the future time-slices (2021–2050 and 2051–2080) from the model ensemble. In Fig. 3, we present only the scenario SSP5-8.5. However, results for the scenarios SSP1-2.6 and SSP3-7.0 are available in the supplementary material (Online Resources 8–13).

Relatively small changes in PRCPTOT are expected for future climate scenarios (Fig. 3a). October is the month with the lowest precipitation values, and the rainy season seems to start in November. The most remarkable future changes occur in January (mostly in SSP5-8.5, 2051–2080), with increasing precipitation, and October, with decreasing precipitation. In general, we can observe that for both sites, shifts in precipitation seasonality may occur, which will reflect in changes in future agricultural practices. In October, the precipitation in Syferkuil may reduce from 50.2 mm (1981–2010) to 42.8 and 35 mm (SSP5-8.5, 2021–2050 and 2051–2080). In Univen, this reduction is from 44.3 mm to 37.4 and 27.6 mm (SSP5-8.5, 2021–2050 and 2051–2080). For November, February, and March, different patterns are found according to the time-slice. In December and January, we identify a trend to increase precipitation in both locations. In December, this increase is from 105.3 mm (baseline) up to 115.7 mm (SS5-8.5, 2051–2080) in Syferkuil, and from 100.8 mm up to 109.2 mm (SSP5-8.5, 2051–2080) in Univen. In January, this increase is from 126.2 mm up to 136.1 mm (SS5-8.5, 2051–2080) in Syferkuil and from 148.1 mm up to 163.5 mm (SSP5-8.5, 2051–2080) in Univen. This will certainly have impacts over the maize yield, as we discuss in the “Impacts of climate change on maize” section.

Regarding the LWP index (Fig. 3b), it is expected decreasing of LWP in north Limpopo in November and south Limpopo in October (mostly in SSP5-8.5, 2051–2080). In December, the increasing or decreasing of LWP depends on the climate scenario evaluated. There is an increase of LWP in the north region (January) and south region (February), according to the scenario SSP5-8.5 in the time-slice 2051–2080. We found the highest values of RX5D in the baseline period in January and February, in the region of Univen and Syferkuil (Online Resource 9). In January, we identified a trend of increasing RX5D in the future (2051–2080). In November, the ensemble indicated that the Northeast of Limpopo tends to get drier in the future. The evaluation of DD suggests increasing DD (online resource 11) in the Northeast of Limpopo, mostly in October and November. Also, it is shown decreasing in DD in the future in the southeast region, mainly in February. The months of October and March are the months with higher DD values (higher than 26 days). The highest LDP in the region in the baseline period is identified in October and March, while the lowest values are in December (online resource 12). The greatest future changes show increasing LDP in the Northeast region, mostly in SSP5-8.5 (2051–2080).

Regarding the SPI index (Fig. 3c), the South and Central regions of Limpopo tend to have more problems related to droughts. In the future, the index indicates increases in droughts in October in the southern region. In November, a decrease in droughts in the northern region and an increase in the south of the region are expected. December is currently the month with the most problems concerning droughts. However, there are different trends for December according to different future scenarios. According to current climate conditions, January is not a month prone to drought. Still, it may become drier in the future, mainly in the southern region of Limpopo, as indicated by SSP1-2.6 and SSP3-7.0 (2051–2080).

We conclude, therefore, that both subregions are likely to have more severe drought conditions in the future than during the baseline period 1981–2010. Other studies, which evaluated different indexes, come to similar conclusions. For example, Gizaw and Gan (2017) used the Palmer drought severity index to analyze changes in droughts, and they concluded that most South African areas would shift to a drier climate in the 2050s and 2080s. The results agree with Schulze et al. (2001), who reported that in SA, which is already a water-stressed country, climate change is expected to increase the variability of rainfall events and amplify weather extremes.

**Fig. 4:**
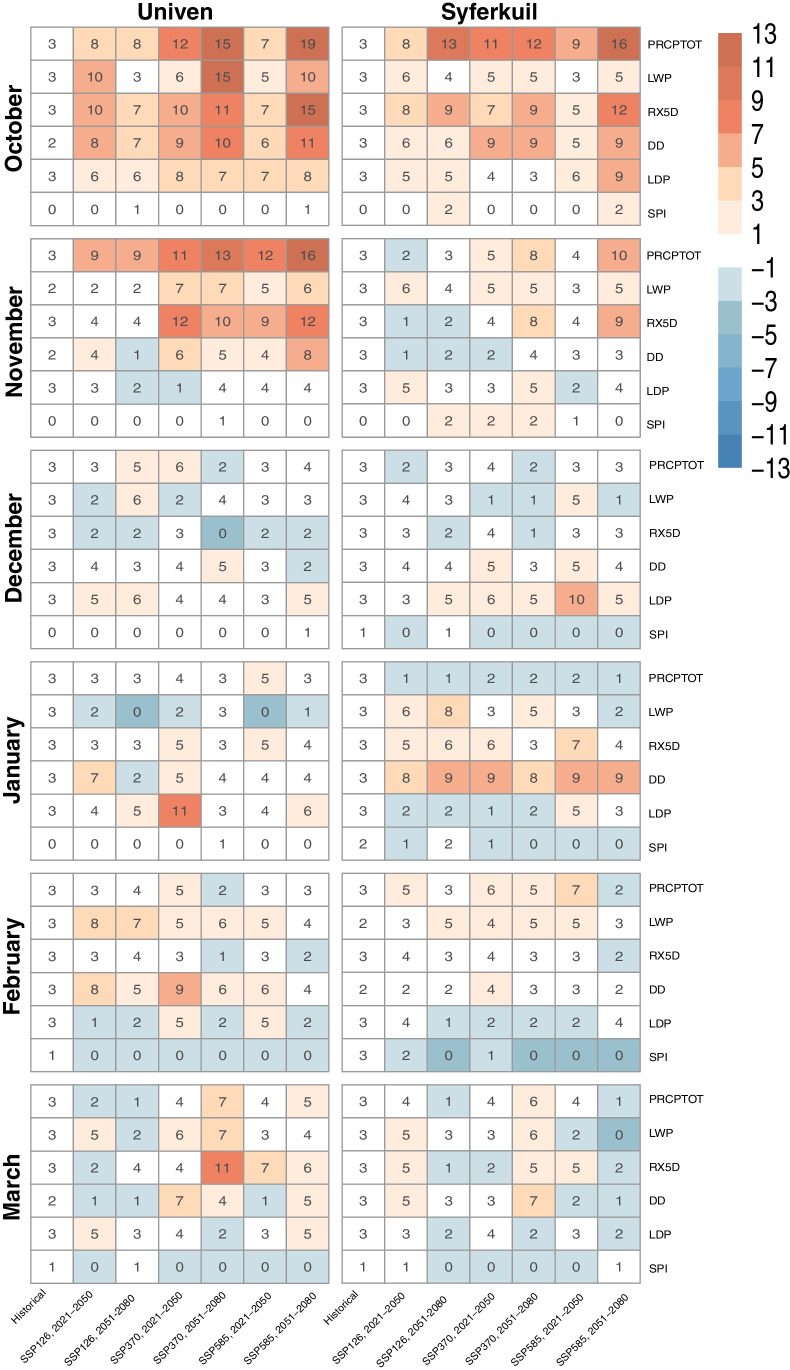
Number of years with drought conditions (DC) in Univen and Syferkuil. Colors indicate the difference between future scenarios and historical simulation. Shades of red show drier conditions in the future compared to historical simulation, and blue shades show wetter conditions in the future

To identify future changes in droughts frequency, we evaluated drought conditions (DC) based on the quantiles thresholds. We calculate the number of years in the future with DC and compare it to the current DC. Figure 4 indicates the number of years with DC in Univen and Syferkuil, according to the historical simulation and SSP's, in different 30-year time-slices. We can see that there are some local differences regarding the frequency of DC (historical and future) at the two sites.

In Univen, the worst DC tends to occur in October, with an increase of DC indicated by all evaluated indices. In the scenario SSP5-8.5 (2051–2081), PRCPTOT presented 19 years with DC, representing an increase of 16 years in the 30-year time-slice (around 53.3%). In the RX5D index, we observe an increase of 12 years, and DD presented an increase of 9 years. There is an agreement in drought patterns among the climate scenarios. There is a decrease in PRCPTOT, LWP, and RX5D and an increase in DD in November. The worst DC also occurs in SSP5-8.5 (2051–2080).

At Syferkuil, the worst future DC occurs in October, although with lower values than Univen. In SSP5-8.5 (2051–2080), results show an increase of 13 years with DC related to PRCPTOT in the 30-year time-slice, 9 years in the RX5D index, and 6 years in DD and LDP indices. In January, we identified a decrease in LWP and RX5D and increasing in DD. Although January is a month with an increasing rainfall trend in Syferkuil, there was also an increase in DC in all scenarios, according to some indices. The DD index shows an increase of DC from 3 years throughout historical simulations to 9 years in a future scenario and RX5D an increase from 3 to 7 years (in the worst-case-scenario).

We conclude that the Univen site represents the subregion with the most remarkable changes, increasing DC in the future. It was also the subregion that presented the worst DC in the historical period, which is a reason for concern, primarily due to the potential impacts of DC on maize production.

### Impacts of climate change on maize

**Fig. 5:**
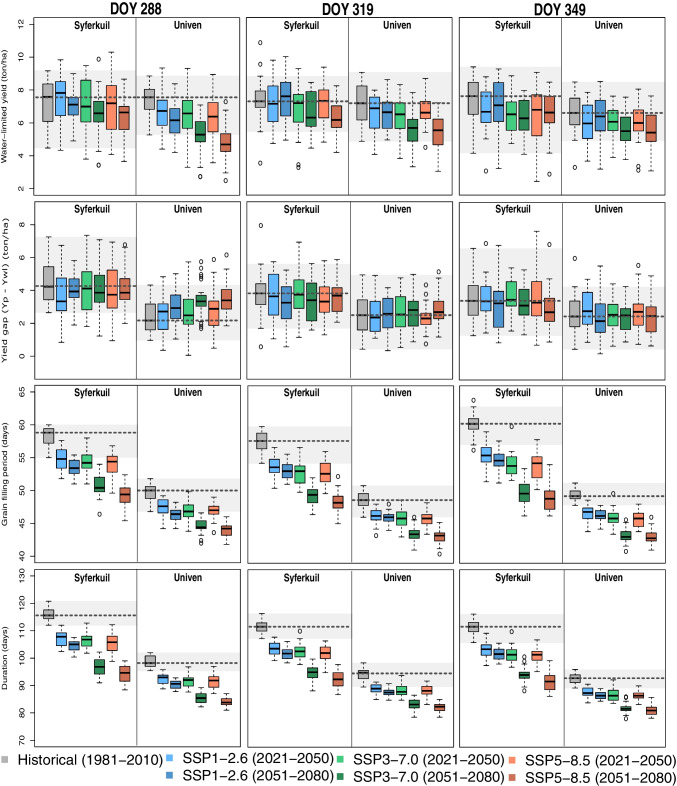
Historical and future water-limited maize yield (t.ha − 1), yield gap (Yp-Ywl) (ton/ha), grain filling period (days), and growth duration (days), in Univen and Syferkuil, according to different emergence dates (15 October (DOY 288), 15 November (319), and 15 December (349) days). The dotted grey lines indicate the median values, and the shade of grey indicates the maximum and minimum values of the historical simulation

Water-limited yield was similar among sites (Fig. 5). However, the future projections for Univen indicate a reduction in yield, regardless of the climate scenario assessed. The highest reduction and lowest yields ​​are found under the SSP5-8.5 (2051–2080) scenario. It is also noteworthy that for baseline yield simulations, the runs with an emergence date at day 288 resulted in the highest yield (~ 7.33 t.ha^−1^), as this coincides with the crop with closed canopy being exposed to high global radiation levels with sufficient moisture in an optimum manner under the given baseline climate. However, in the scenario SSP5-8.5 (2051–2080), this start date led to the worst yield performance (~ 4.88 t.ha^−1^), which is likely due to shifts in the rainfall season, as can be derived from negative changes in drought indices (Figs. 3 and 4) at the start of the rainy season under future conditions. In Fig. 4, we highlighted that the total amount of precipitation in October tends to decrease, regardless of the climate scenario, in both sites. In December and January, we expect an increase in future precipitation. Due to this future shift in the rainy season, a delay in the emergence date seen to be a good strategy to cope with climate changes and maintain reasonable yields. The lowest yield reduction in SSP5-8.5 is found in runs with an emergence date of 349 days, which can also be related to the smaller future changes in total precipitation in December (Figs. 3 and 4).

Mangani et al. (2018) evaluated two versions of the crop model CropSyst to simulate crop yield in SA and concluded that in climate change scenarios (2030 and 2050), a decrease in maize yield is expected due to the increase of drought severity. However, the understanding of the period when these droughts will be more severe is also important to create mitigation and adaptation measures under climate change scenarios. By comparing the simulated yields and their changes under future conditions with those of the drought indices (Fig. 5), we derive that in the future, drier conditions in October may strongly affect the yield of the Univen region, with the worst scenarios for early sowing and the period 2051–2080 (scenarios SSP3-7.0 and SSP5-8.5). A possible alternative could be to delay the sowing and emergence dates to November or December to reduce yield losses. Considering the SSP5-8.5 scenario (2051–2080) as an example, the yield values ​​at Univen differ considerably depending on the crop emergence date: between 4.88 t.ha^−1^ (DOY 288) and 5.41 (319) up to 5.55 (349). At Syferkuil, most yield simulations for future conditions also indicate reduced yield, with the worst scenarios in 2051–2080 (SSP3-7.0 and SSP5-8.5). For the baseline period, the yield variability is smaller. We conclude that shifting emergence dates under future conditions can considerably impact yield at both sites significantly by taking into account shifts in seasonality and adjusting accordingly by later sowing to reduce potential yield losses.

The highest values of yield losses found in Syferkuil and Univen were in SSP5-8.5 (2051–2080). In Syferkuil, these losses were − 15.5% (considering the EM 288), − 18.6% (319), and − 10.7% (349). In Univen, the yield losses were − 50.3% (288), − 31.2 (319), and − 19.2 (349). The situations that could represent an increase were found in Syferkuil (SSP1-2.6), with a yield increase of 2.9% (2021–2050, EM 288) and 1.6% (2051–2080, EM 319).

The differences between simulated potential land water-limited yields can serve as an indication of the degree of long-term average water-limitation and how that shifts under climate change scenarios and alternative sowing/emergence date. The simulations show that Univen has lower differences between potential yield and water-limited yield than Syferkuil, and these values do not have a significant relationship with emergence dates. In summary, these differences tend to decrease in Syferkuil and increase in Univen. Regarding the grain filling period, Univen presented fewer days than Syferkuil. This duration tends to decrease in both sites in the future, with the worst scenarios in 2051–2080 (SSP3-7.0 and SSP5-8.5). The cycle duration is higher when EM is 288 in both sites, although the differences between EM are not high. The site Syferkuil has a higher cycle duration than Univen, although both areas indicate a decrease in cycle duration in future scenarios, mainly in SSP3-7.0 and SSP5-8.5 (2051–2080).

The occurrence of drought events in the future may affect yield and climate change adaptation, or more generally future management practices. Assessing the period in which droughts occur is essential as it can affect different maize growth stages, causing damages or sub-optimum growth conditions that call for specific adaptations. When maize is exposed to drought conditions during the vegetative stage, yield losses can reportedly range from 32% to as high as 92% (Atteya 2003). Other authors have found yield losses for the reproductive stage or early grain filling ranging from 63 to 87% (Kamara et al. 2003) and for the late grain-filling and ripening period from 79 to 81% (Monneveux et al. 2006). The occurrence of other agroclimatic extreme events also threatens food security as they may affect food crop production worldwide (see, e.g., Rötter et al. 2018). Mangani et al. (2019) used climate change scenarios and crop models to study potential climate change impacts and concluded that maize yield is expected to be reduced in the future (2051–2080) in SA. Similar results were reported by Cammarano et al. (2020) for commercial maize farming in the free state of SA. Masupha and Moeletsi (2018) used drought indicators to study how future droughts may limit maize production in SA and concluded that drought remains a threat to rainfed maize production in the Luvuvhu River catchment area.

Climate-induced changes in productivity due to droughts are already perceived by farmers in the Mopani district of the Limpopo Province. In her master thesis, May (2019) conducted a survey and concluded that most farmers from the four villages surveyed (Ndengeza, Makhushane, Mafarana, and Gabaza) who perceived changes in the climate over the past decade and increased frequency of extreme years also reported negative effects on their maize yields. The survey also confirmed that in Mafarana, Gabaza, and Ndengeza, for most farmers, October and November is the usual sowing period. In the drier village Makhushane, most farmers are sowing their maize later, in November and December.

Some improved agro-technologies (seasonal weather forecast-based sowing; more drought-tolerant maize cultivars) and management practices (combinations of sowing date and cultivar choice depending on the onset of rains) in the future could be utilized to minimize the impacts of droughts in future maize production. This would require still higher investments in climate information services and in breeding climate-resilient maize cultivars using advanced breeding tools (Rötter et al. 2015; Cairns et al. 2018; Hoffmann et al. 2018) and/or the judicious and site-specific choice of climate-smart interventions, such as cereal-legume intercropping and crop rotations (Swanepoel et al. 2018; Rapholo et al. 2019; Hoffmann et al. 2020).

## Conclusions

We aimed to characterize drought patterns and to evaluate their spatiotemporal variability and potential impacts on maize production in the Limpopo province with a closer look at two different climatic subregions. Climate models and drought indicators were used to quantify droughts and changes in their frequency in the future. This was then linked to the quantification of yield impacts for different sowing/crop emergence dates using the climate data in conjunction with the dynamic crop simulation model WOFOST.

The key messages of this research are as follows:

- Current drought conditions (DC): Compared to Syferkuil, Univen showed the driest conditions of PRCPTOT. October appears as the month with the worst drought conditions, considering PRCPTOT, LWP, and RX5D. In Syferkuil, October is also the month with the worst DC indicated by DD and LDP. In Univen, DD and LDP have the worst DC in March.
- Climate models performance: The estimation of drought indices with a model ensemble was better than those with individual models. The patterns of underestimation or overestimation depend on the index studied, the model evaluated, and the region.
- Historical and future drought patterns: The climate scenarios indicate small changes in the future for the PRCPTOT index. Drought indices indicated that mainly in the scenario SSP5-8.5 (2051–2080), Univen and Syferkuil will present worse DC in the future.
- Historical and future frequency of droughts: The worst DC tends to occur in October, considering all the evaluated indices. Univen site was the site with the greatest future changes, with the increasing of DC.
- Drought’s impacts on maize production: The yield tends to decline in the future considering all emergence dates. We conclude that future changes in the emergence date seem to impact yield in both sites significantly. A possible alternative is to delay the emergence date to November or December to reduce the yield losses. The grain filling period, as well as the cycle duration, tends to decrease in the future. The cycle duration is higher when EM is 288 in both sites.

Understanding historical drought patterns and the future perspective is important for the implementation of drought plans and mitigation measures, to promote sustainable management options, and to support crop ideotype design. Current and future drought conditions in October indicate that droughts will increase in this period, mostly in the mid-end century. The increase in future drought conditions will have a direct impact on maize production, representing a risk for food security in the region. The results found in this paper can contribute to specific measures to support improvement of maize production in SA, considering changes in future drought patterns and their effect on yield, grain filling, and cycle duration.

## Supplementary Information

Below is the link to the electronic supplementary material.

Supplementary file1 (PDF 37124 KB)
