# Supplementary material for: Drought patterns: their spatiotemporal variability and impacts on maize production in Limpopo province, South Africa
Source: Int J Biometeorol. 2022 Dec 7;67(1):133–48. doi: 10.1007/s00484-022-02392-1 (PMC9758106; doi:10.1007/s00484-022-02392-1)
Supplement: Supplementary file 1 — Supplementary file1 (PDF 37124 KB) [file 484_2022_2392_MOESM1_ESM.pdf]

Supplementary material

Online Resource 1. Coefficient of variation (%) of observed temperature and precipitation (1984-2014) in Syferkuil and Univen

	Temperature	Precipitation
Syferkuil	22.2	119.9
Univen	14.1	139.4

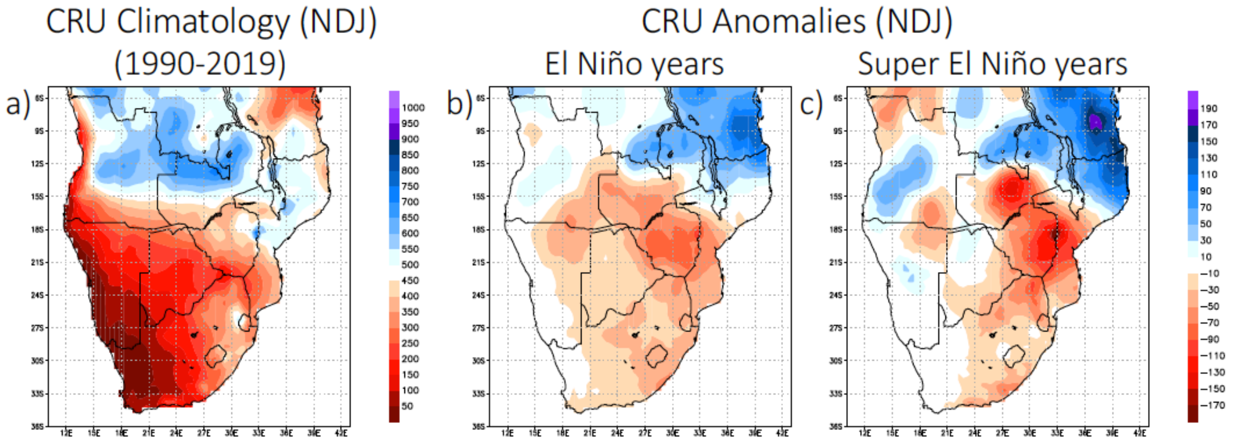

Online Resource 2. (a) Climatic Research Unit gridded Time Series (CRU TS) (Harris et al. 2020) precipitation climatology (1990-2019, NDJ) and the anomalies of precipitation (in mm) in (b) El Niño years and (c) Super El Niño years, according to the Oceanic Niño Index (ONI) (Huang et al. 2017)

.

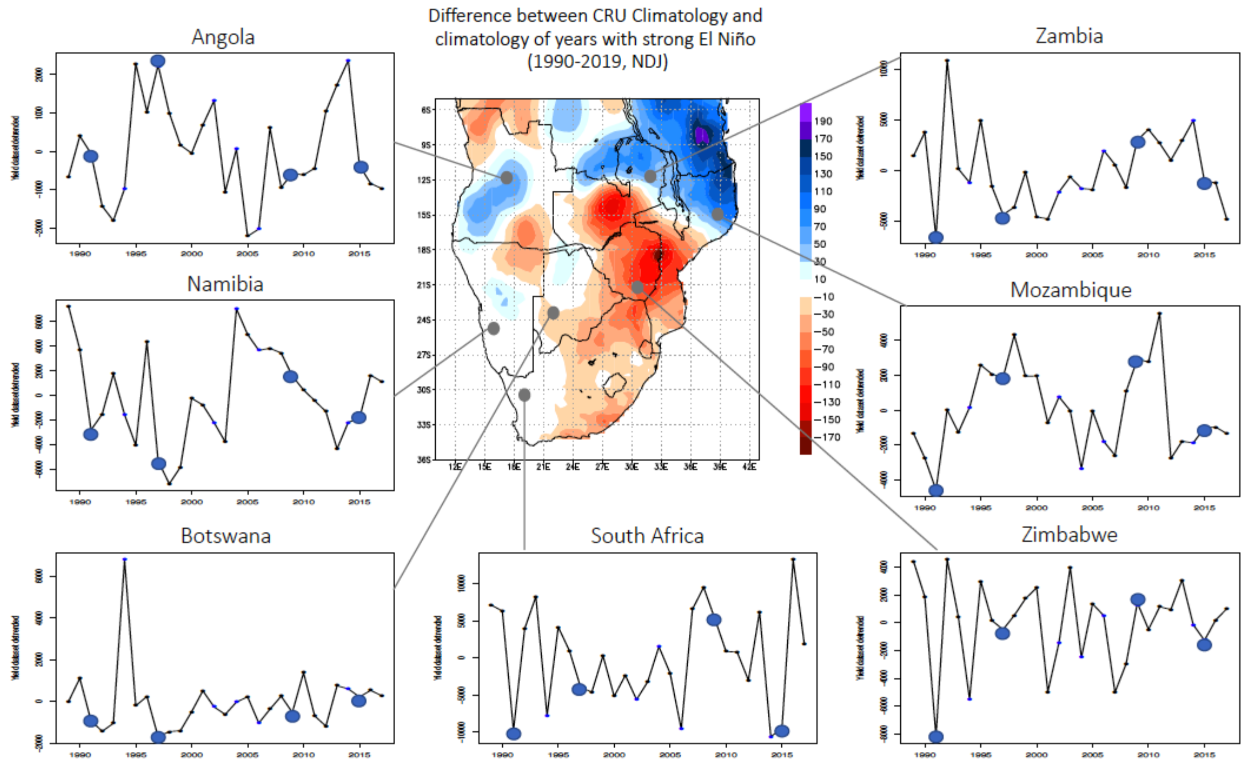

Online Resource 3. Difference between the Climatic Research Unit gridded Time Series (CRU TS) (Harris et al. 2020) precipitation climatology (1990-2019, NDJ) and the climatology in strong El Niño years (in mm). The adjacent figures represent the yield dataset (FAOSTAT) detrended (t/ha). Blue points indicate years with El Niño, and larger points indicate strong El Niño occurrence, according to the Oceanic Niño Index (ONI) (Huang et al. 2017)

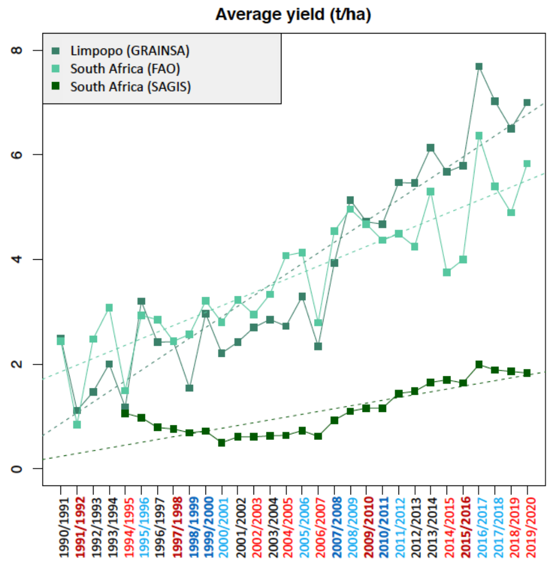

Online Resource 4 Average maize commercial yield (t/ha) in Limpopo (GRAINSA, 1990-2020) and South Africa (FAO, 1990-2020), and non-commercial yield in South Africa (SAGIS, 1994-2020), from 1990 to 2019. Years colored in red (blue) refers to El Niño (La Niña) occurrence, according to the Oceanic Niño Index (ONI). Years in bold indicate strong El Niño/La Niña event

Online Resource 5. Set up run and WOFOST parameters.

General	Number of successive production levels	Simulation of potential growth and simulation of water-limited crop growth
	Water-limited crop growth	Effects of drought only
	Summary seasonal soil water balance	Whole system and root zone
Crop	Crop file	Maize (Van Heemst, 1988)
Timer	**General timer**	
	Output interval days	1
	Start date water balance	1
	**Weather time**	
	Selected stations	Univen; Syferkuil (grid boxes containing stations)
	Available years	Simulated historical (1981-2010)<br>SSP1-2.6; SSP3-7.0; SSP5-8.5 (2021-2050; 2051-2080)
	Start year	1981; 2021; 2050
	Consecutive years	30
	**Crop timer**	
	Selected crop	Maize (Van Heemst, 1988)
	**Start day**	
	Fixed emergence date	15-Oct (DOY 288)<br>15-Nov (DOY 319)<br>15-Dec (DOY 349)
	**End day**	
	Maturity (<= max duration)	Maximum duration 260 days
Soil	**Hydrology**	
	Initial surface storage (cm)	0
	Maximum surface storage (cm)	0
	Initial available soil water (cm)	10
	Maximum initial moisture content in initial rooting depth (-)	0.099 – 0.104
	Maximum rooting depth of soil (cm)	100
	Non-infiltration fraction	Fixed
	Maximum non-infiltrating factor of rain storm	0

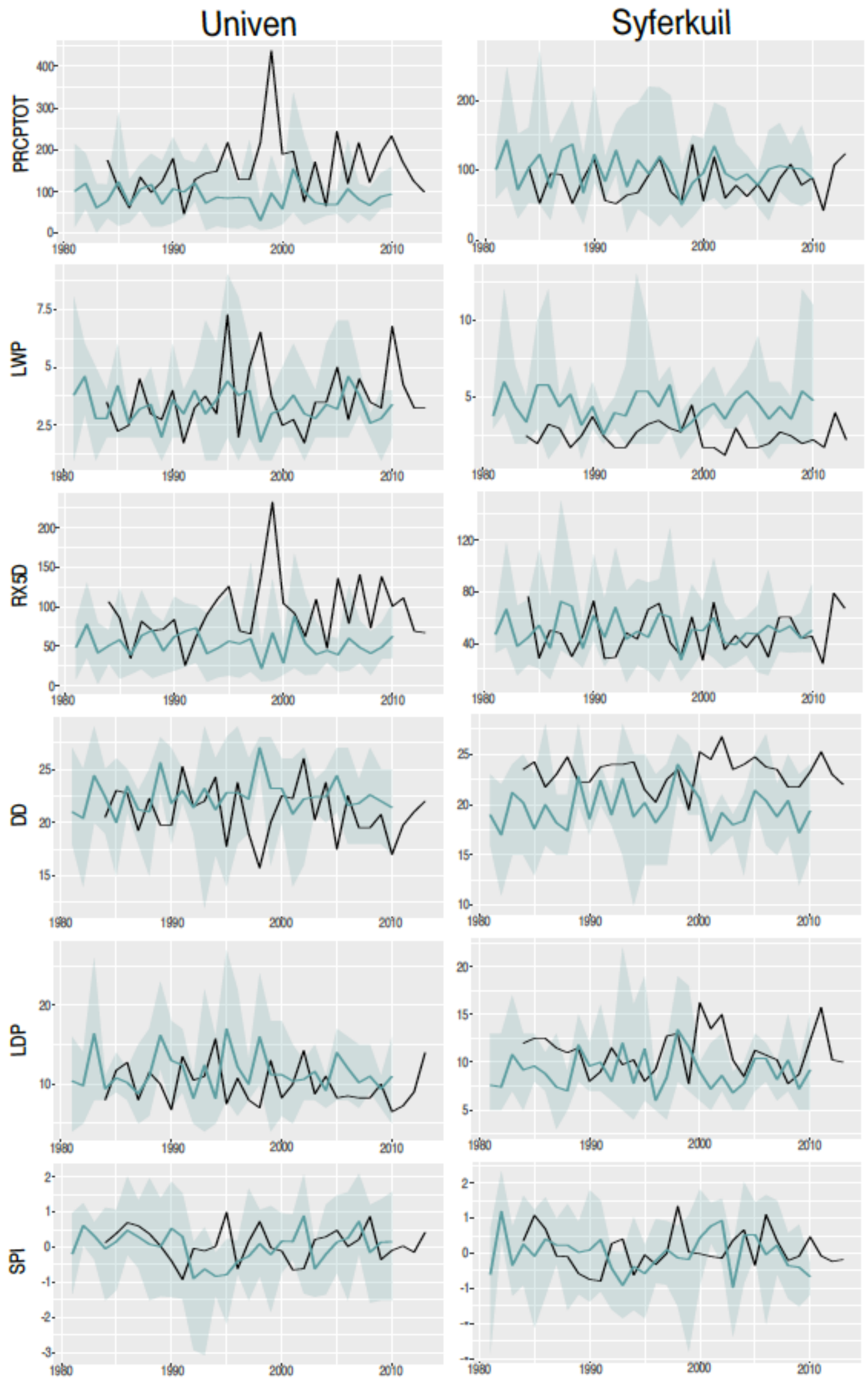

Online Resource 6. Drought climatology using observed data (1984-2013, black line) and climate models ensemble data (1981-2010, blue line). The range of maximum and minimum values of simulated members are represented with the blue shade.

Online Resource 7. Drought indices for the historical period (1981-2010, model ensemble). Red and blue colors indicate the worst and best conditions, respectively.

Drought index	Quantile	Univen						Syferkuil					
		Oct	Nov	Dec	Jan	Feb	Mar	Oct	Nov	Dec	Jan	Feb	Mar
PRCPTOT	q < 0.1	**28.4**	67.0	70.4	**94.7**	59.5	34.3	**35.7**	74.2	76.4	81.6	**84.0**	48.8
LWP	q < 0.1	**2.0**	2.6	**3.0**	2.8	**3.0**	**2.0**	**2.2**	**3.4**	3.2	**3.4**	**3.4**	2.6
RX5D	q < 0.1	**17.2**	39.5	37.1	**55.5**	40.3	26.8	**19.5**	37.7	38.7	**51.0**	44.9	30.2
DD	q > 0.9	27.2	24.4	24.3	24.2	**22.6**	**27.4**	**26.0**	22.4	22.3	22.5	**21.6**	25.4
LDP	q > 0.9	18.6	16.0	**13.8**	14.2	15.9	**19.3**	**17.2**	11.6	**11.0**	13.5	13.2	16.2
SPI	-	-1.0	-1.0	-1.0	-1.0	-1.0	-1.0	-1.0	-1.0	-1.0	-1.0	-1.0	-1.0

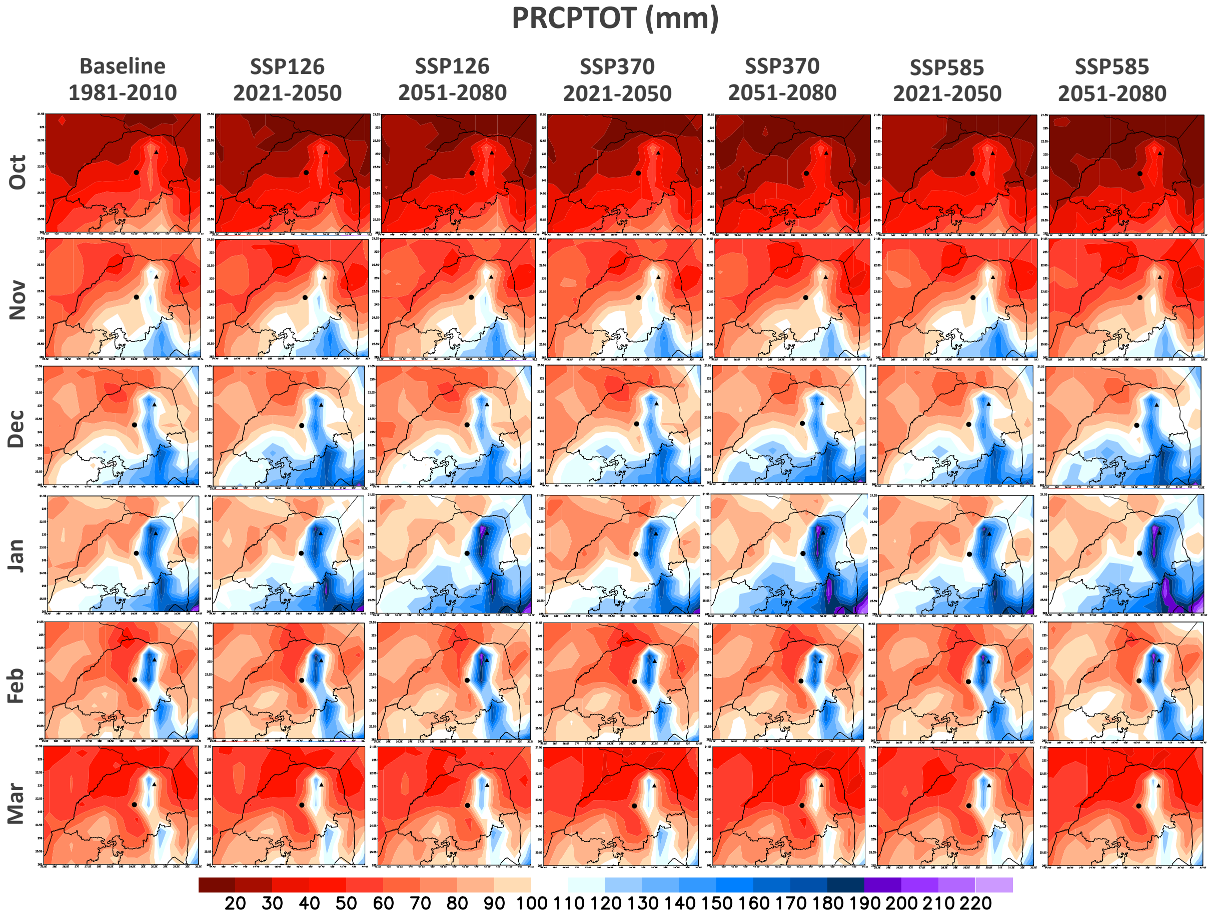

Online Resource 8. Ensemble 30-year monthly average PRCPTOT in the Limpopo province, for the growing season months October to March (rows). The columns indicate historical simulations and future scenarios projections for different periods. The circle represents Syferkuil, and the triangle Univen.

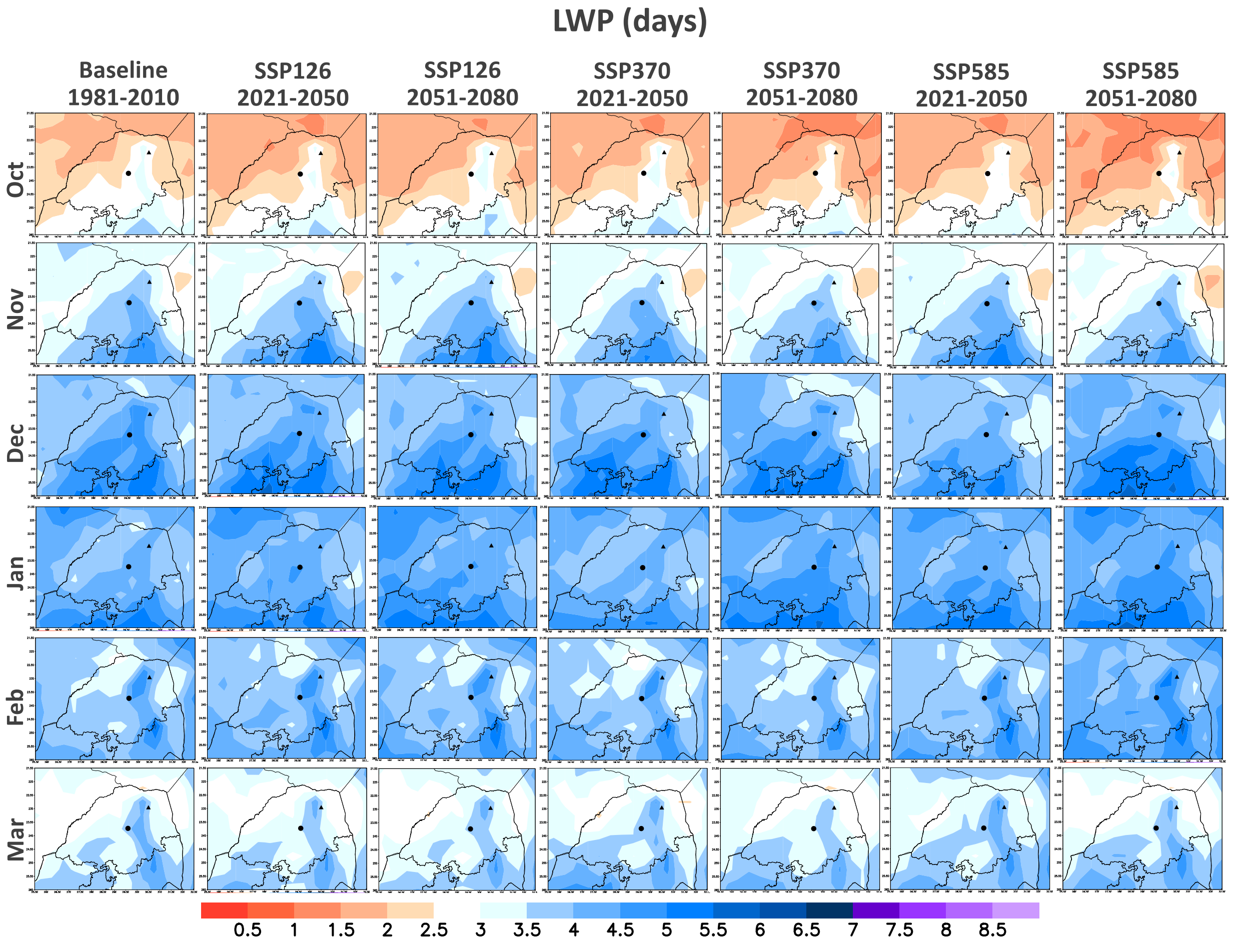

Online Resource 9. Ensemble 30-year monthly average LWP in the Limpopo province, for the growing season months October to March (rows). The columns indicate historical simulations and future scenarios projections for different periods. The circle represents Syferkuil, and the triangle Univen.

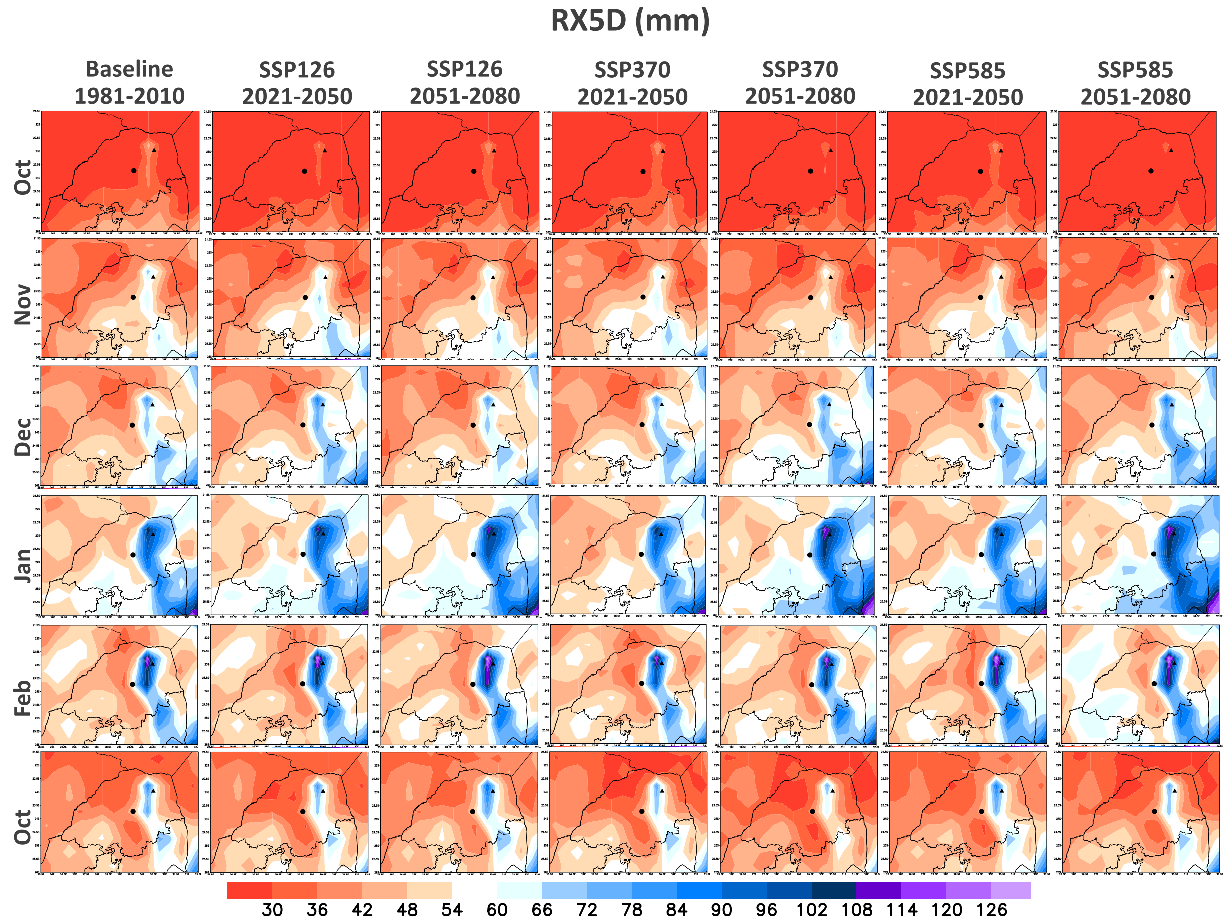

Online Resource 10. Ensemble 30-year monthly average RX5D in the Limpopo province, for the growing season months October to March (rows). The columns indicate historical simulations and future scenarios projections for different periods. The circle represents Syferkuil, and the triangle Univen.

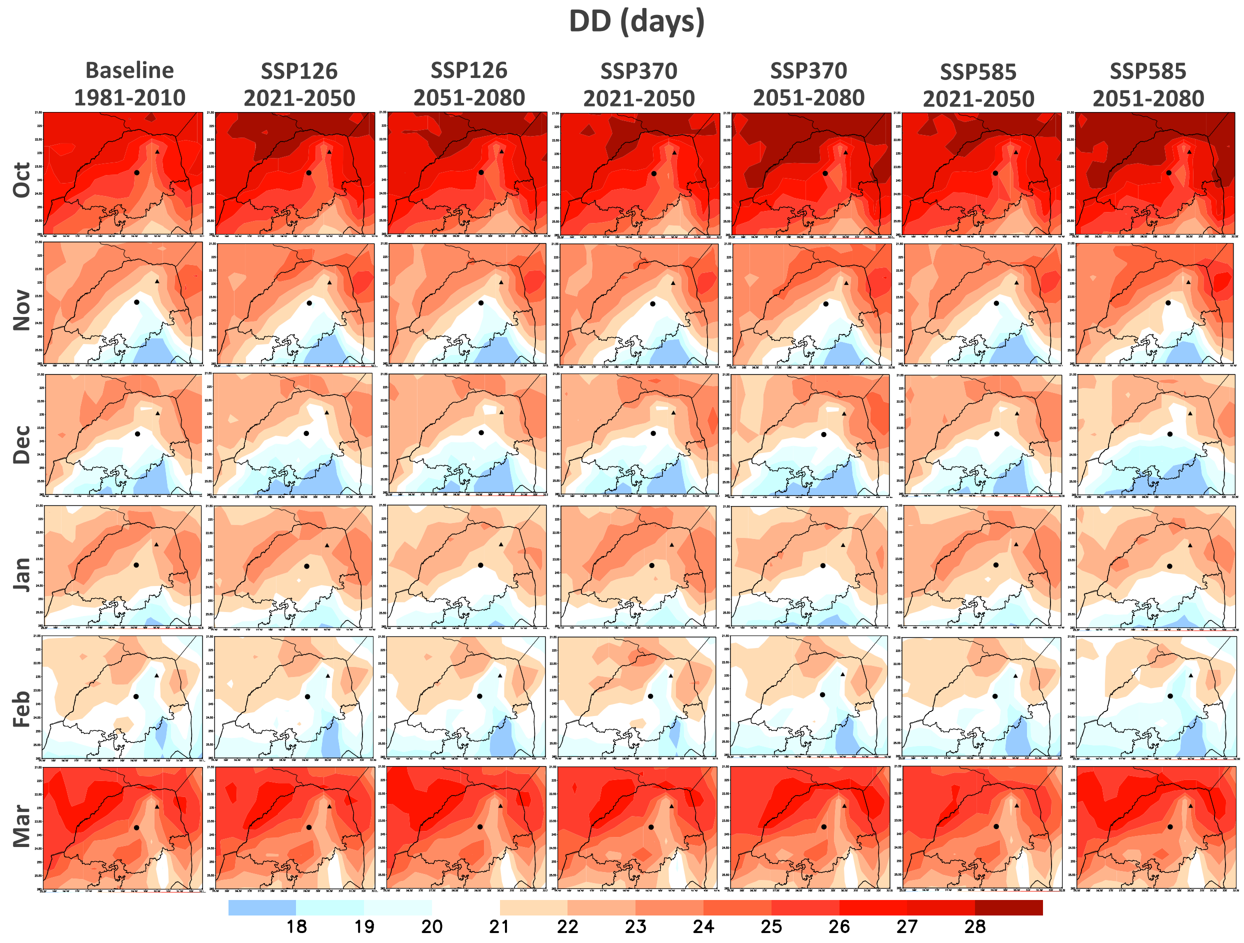

Online Resource 11. Ensemble 30-year monthly average DD in the Limpopo province, for the growing season months October to March (rows). The columns indicate historical simulations and future scenarios projections for different periods. The circle represents Syferkuil, and the triangle Univen.

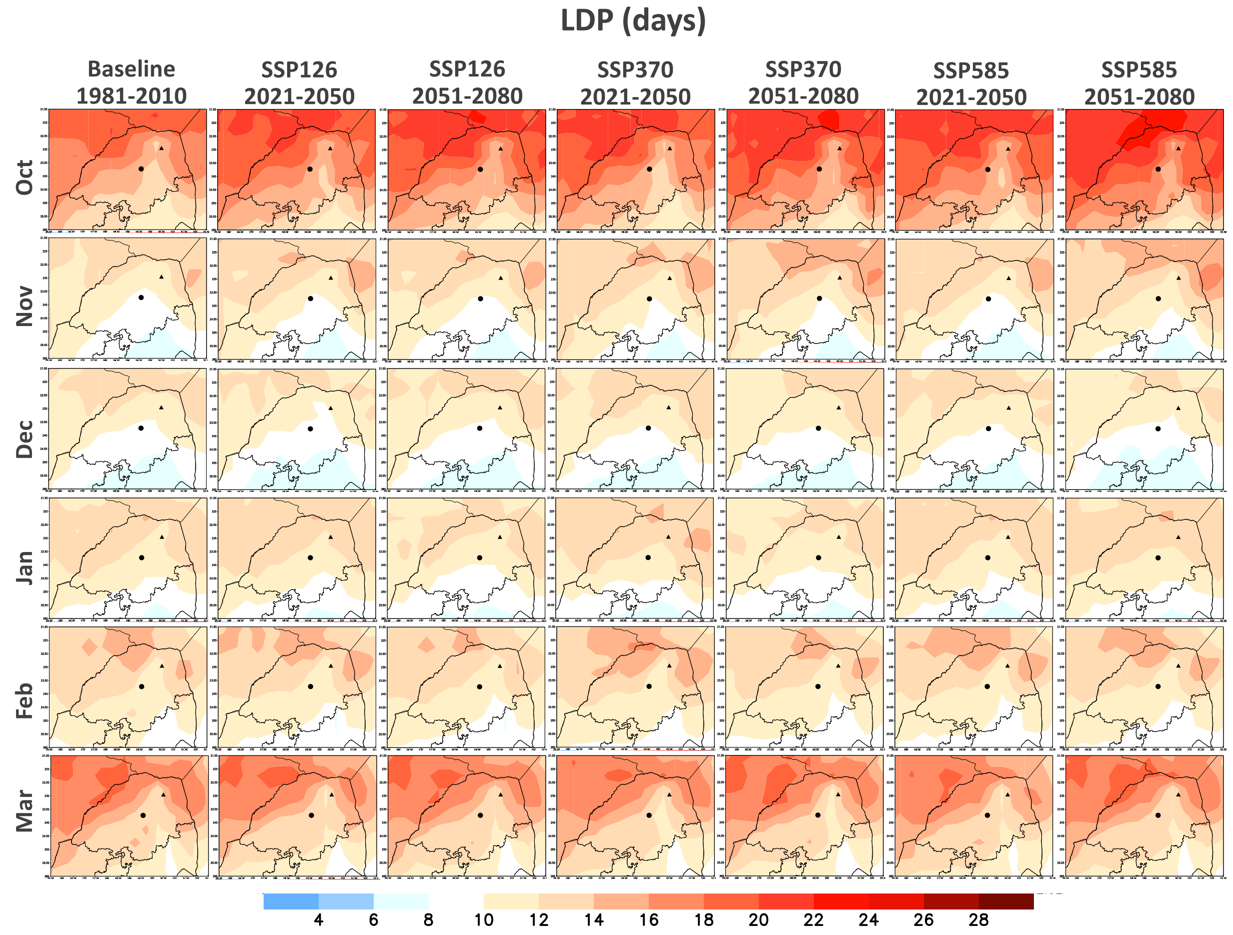

Online Resource 12. Ensemble 30-year monthly average LDP in the Limpopo province, for the growing season months October to March (rows). The columns indicate historical simulations and future scenarios projections for different periods. The circle represents Syferkuil, and the triangle Univen.

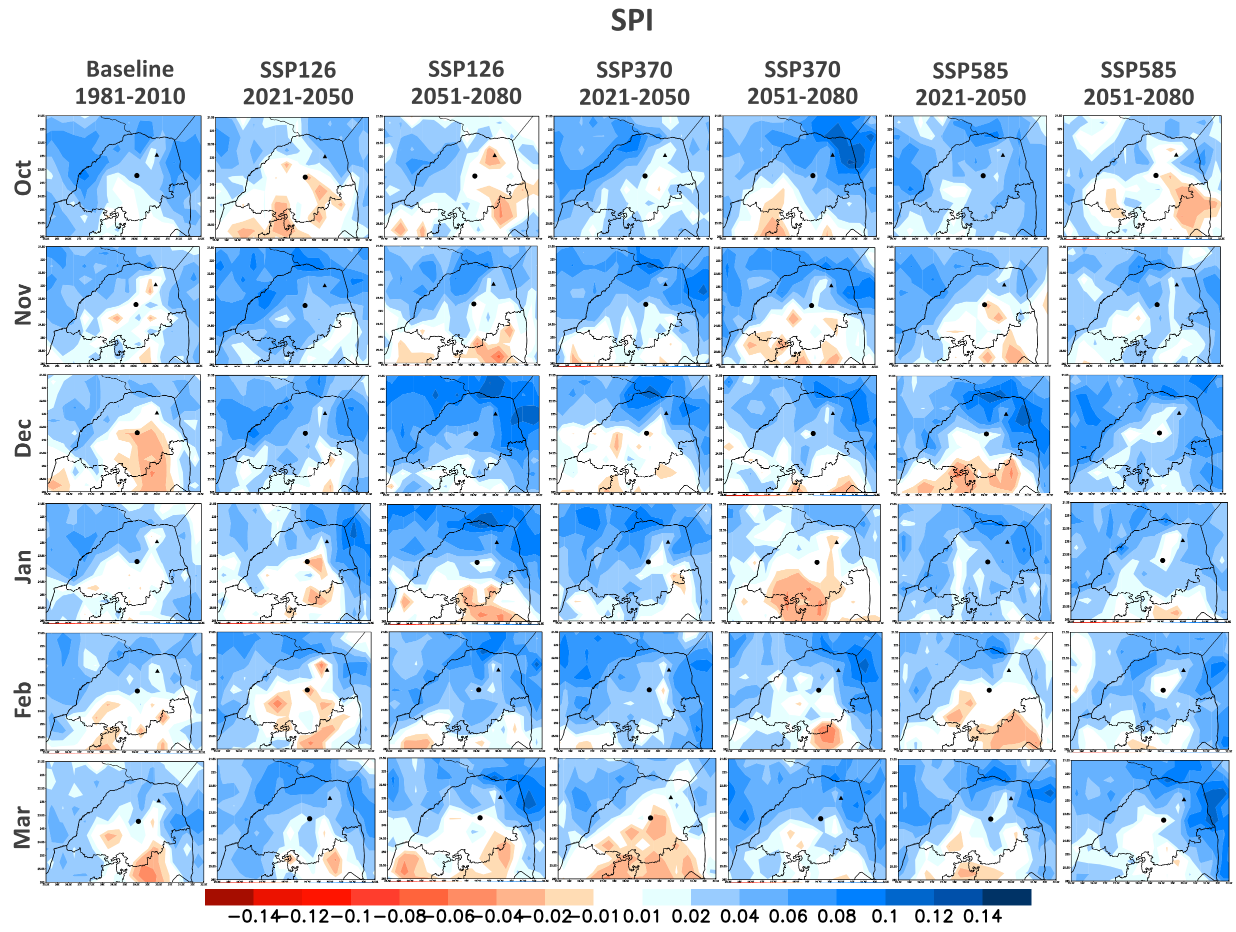

Online Resource 13. Ensemble 30-year monthly average SPI in the Limpopo province, for the growing season months October to March (rows). The columns indicate historical simulations and future scenarios projections for different periods. The circle represents Syferkuil, and the triangle Univen.

References

Harris, I., Osborn, T. J., Jones, P., & Lister, D. (2020). Version 4 of the CRU TS monthly high-resolution gridded multivariate climate dataset. Scientific data, 7(1), 1-18.

Huang, B., Thorne, P. W., Banzon, V. F., Boyer, T., Chepurin, G., Lawrimore, J. H., ... & Zhang, H. M. (2017). Extended reconstructed sea surface temperature, version 5 (ERSSTv5): upgrades, validations, and intercomparisons. Journal of Climate, 30(20), 8179-8205.

O'Neill, B. C., Carter, T. R., Ebi, K., Harrison, P. A., Kemp-Benedict, E., Kok, K., ... & van Ruijven, B. J. (2020). Achievements and needs for the climate change scenario framework. Nature Climate Change, 1-11.

Van Heemst, H. D. J. (1988). Plant data values required for simple crop growth simulation models: review and bibliography. Simulation Reports CABO-TT No. 17. Centre for Agrobiological Research and Department of Theoretical Production Ecology, Agricultural University. Wageningen, the Netherlands, 100 pp.
